# Characteristics, surgical treatment, and outcomes of injuries involving the tarsus in greyhounds

**DOI:** 10.3389/fvets.2023.1234206

**Published:** 2023-08-08

**Authors:** Morgan R. Biggo, Stephen C. Jones, Audrey W. Wanstrath, Selena Tinga, Jonathan Dyce, Brittney A. Carson, Kelsey Schaul, Christelle M. Follette, Nina R. Kieves

**Affiliations:** Department of Veterinary Clinical Sciences, College of Veterinary Medicine, The Ohio State University, Columbus, OH, United States

**Keywords:** greyhound, tarsus, injury, surgical site infection, explantation

## Abstract

**Objective:**

The first objective of this study was to describe the type of tarsal injuries sustained, surgery performed, and postoperative complications in greyhounds presenting to a single veterinary hospital. An additional objective of the study was to determine the surgical site infection (SSI) and explantation rate, and if any variables were associated with an increased risk of SSI and/or explantation.

**Animals:**

116 greyhounds receiving surgical intervention for a tarsal injury.

**Proceures:**

Medical records from a single veterinary referral hospital were reviewed retrospectively. Data retrieved included signalment, details regarding the injury, surgical intervention, concurrent castration, surgical/anesthesia times, postoperative management, time to healing, and postoperative complications. In cases that underwent explantation, cause, time from initial surgery, and risk factors were evaluated.

**Results:**

The most frequently diagnosed tarsal injuries were fracture of the central tarsal bone (CTB; 57.8%), calcaneal fracture (56.9%) and proximal intertarsal subluxation (34.5%). The most common injury combination was a CTB fracture with a calcaneal fracture (31.9%). In total 115 (99.1%) survived to discharge. Of these, 46 (40.0%) were diagnosed with an SSI and 59 (51.3%) underwent explantation. The most common indication for explantation was SSI. Concurrent medial and lateral surgical approaches was found to be associated with an increased likelihood of SSI and explantation.

**Clinical relevance:**

Practitioners performing surgical intervention for tarsal injuries in greyhounds should be aware of the high SSI rate and likelihood that explantation will be required. This risk is elevated for injuries requiring a bilateral surgical approach.

## 1. Introduction

Orthopedic injuries are a common occurrence in the racing greyhound and typically occur by a failure of the musculoskeletal structures to withstand the forces developed during a race (1). In the United States, greyhounds race exclusively in an anticlockwise direction on an oval track, resulting in asymmetric adaptive remodeling of both the skeleton and soft tissues, particularly the left front and right hindlimb (2–4), as the left forelimb acts as a pivot and the right hind limb provides propulsive effort when rounding bends (5). In other countries, they may race clockwise, or both directions. An injury rate of 3.4% was previously reported in racing greyhounds in the United States (6). Tarsal injuries accounted for 6% of total racing injuries with approximately 60% of all tarsal injuries being fractures. Central tarsal bone (CTB) fractures are the most commonly reported tarsal fracture, and numerous other tarsal bone fractures have been reported (7). Complications reported with surgical repair of tarsal fractures include infection, dehiscence, implant failure, implant loosening, delayed union, reduced range of motion, non-union, infective arthritis, and residual lameness secondary to implant irritation of soft tissue (1, 8–13). Many of these complications result in the necessity for additional surgical intervention with added patient morbidity and financial burden. Surgical site infections (SSIs) remain a frustrating and expensive complication in veterinary surgery (14). To the authors’ knowledge, there is no literature investigating the SSI and explantation rate in greyhounds undergoing tarsal surgery. As these injuries often occur in racing greyhounds, financial burden plays a role in their clinical management. Establishing what variables, if any, are associated with an increase in SSI and explantation rate is a first step in minimizing the cost of care and patient morbidity.

There has been no recent literature reviewing common tarsal fracture presentations and postoperative complications in greyhounds. The first objective of this retrospective study was to describe the type of tarsal injuries sustained, surgical intervention performed, and postoperative complications in the unique population of greyhounds presenting to a single referral veterinary medical center. An additional objective of the study, was to determine if any epidemiologic or intraoperative factors were associated with an increased risk of SSI and/or explantation. Based on our experience, we hypothesized that the SSI rate for greyhounds undergoing tarsal surgery would be higher than both the previously reported SSI rate for orthopedic surgery.

## 2. Materials and methods

### 2.1. Study design

Medical records from September 2008 to November 2019 from (location masked for blind review) were reviewed retrospectively for greyhounds undergoing surgical repair of a tarsal injury. Data was collected on signalment, date of injury, nature of injury (racing vs. other), time to surgery from initial injury, lateralization of injury, injury description (bones fractured, open vs. closed fracture, joint luxation), surgical procedure(s) performed, use of bone graft, use of tourniquet, concurrent castration or ovariohysterectomy, surgical time, anesthesia time, primary surgeon (surgical resident vs. board-certified surgeon), postoperative medications, use of bandage/external coaptation, time to discontinuation of bandage/external coaptation use, development of bandage morbidity, time to radiographic healing, evidence of infection, and cause of and time to explantation from initial surgery when performed. Postoperative infection was diagnosed based on positive culture result when available or physical exam (soft tissue edema, incisional drainage, draining tract(s), pain, heat) and radiographic findings (irregular periosteal response, peri-implant radiolucency, implant loosening).

Inclusion criteria included diagnosis of a tarsal injury at presentation for which surgical intervention was performed. Tarsal injuries were diagnosed via history, clinical signs, physical examination, and radiographic findings. All surgical procedures were performed at (location masked for blind review) with each dog having immediate postoperative radiographs available for review. Exclusion criteria included tarsal injuries managed conservatively or injuries not isolated to the tarsus or tibiotarsal joint.

### 2.2. Tarsal injury description

Tarsal injury description was acquired via physical examination and radiographic findings. CTB fractures were graded according to a previously described classification system (Table 1) (15). Fracture of the plantar process of the CTB was recorded separately. Calcaneal fractures were described as simple midbody, comminuted, associated with plantar tarsal ligament avulsion, midbody fracture with fissure extending to the distal articular surface, or base/articular injuries.

**Table 1:** Central tarsal bone fracture classification system as described by Dee et al. (15).

Grade	Central tarsal bone fracture configuration
I	Dorsal slab fracture without displacement
II	Dorsal slab fracture with displacement
III	Dorsal or medial slab fracture with displacement
IV	Both dorsal and medial slab fractures
V	Severely comminuted and displaced fractures

### 2.3. Surgical procedure

The choice of surgical intervention was made on the basis of type of tarsal injury, previously described repair techniques (1, 9, 16–18) and surgeon preference. For each patient undergoing surgical intervention, the following was documented: procedure type, whether a medial and/or lateral approach was performed, if an arthrodesis was performed and if a tourniquet was used. If a CTB fracture was repaired, the screw type(s) used was recorded; additionally, it was noted if the screw was placed in lag or positional technique. If a lateral plate was used to address a calcaneal fracture or joint subluxation/luxation, the type and size of plate used, whether the 4th tarsal bone was engaged through the plate, number of locking screws in the proximal and distal segments, number of cortical screws in the proximal and distal segments, percentage of metatarsal length spanned, and total number of metatarsal cortices captured were recorded. If a bone graft was performed, the timing of the graft (at initial procedure or delayed), as well as location of the graft donor site, were recorded.

### 2.4. Statistical analysis

**Figure 1:**
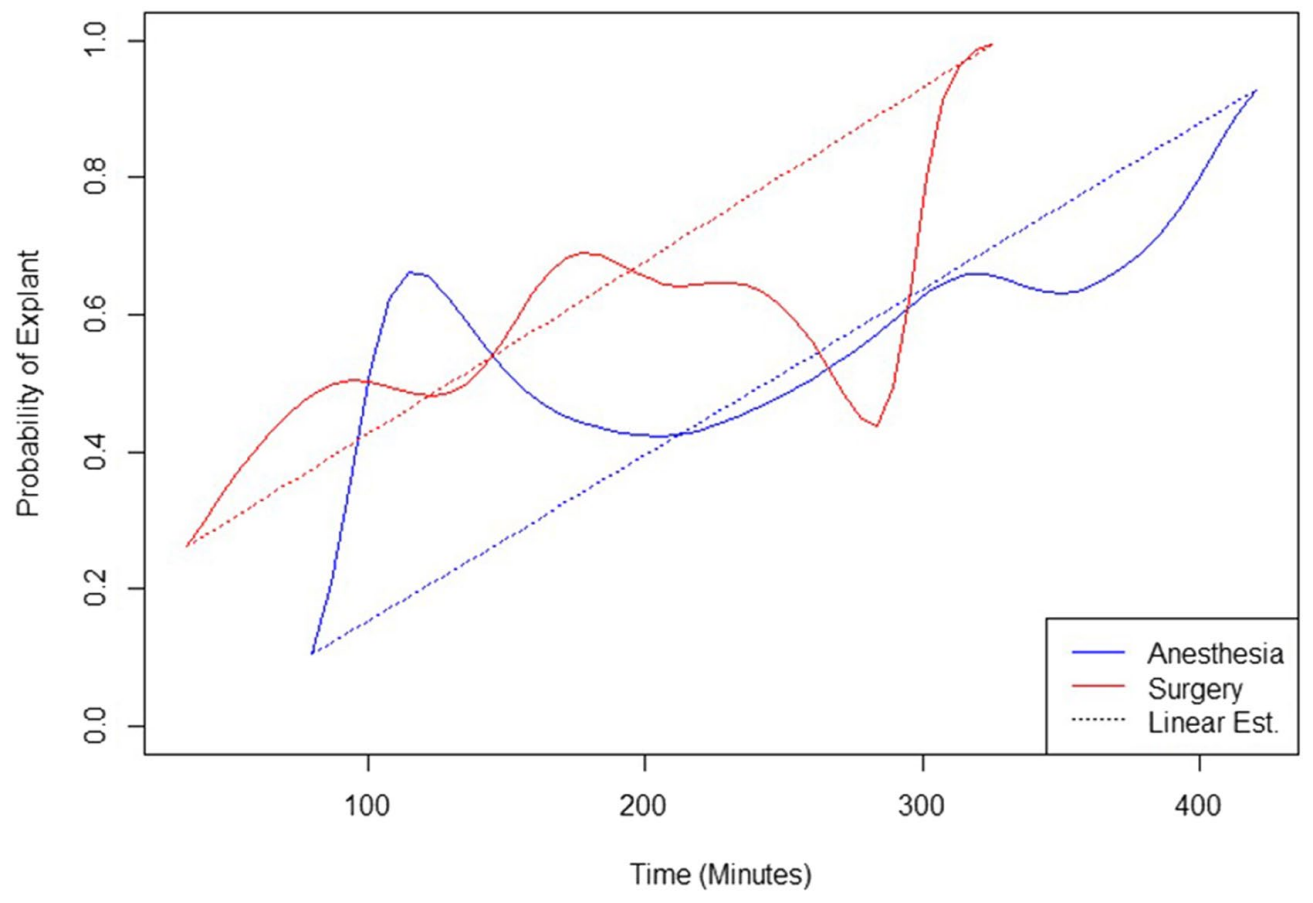
Probabilities of explantation based on anesthetic and surgery times.

All analyses were performed using R version 4.2.2 [(2022-10-31 ucrt)—“Innocent and Trusting” Copyright (C) 2022 The R Foundation for Statistical Computing]. Univariate logistic regressions were used to look at the effect of tarsal injury type, surgical procedures, surgical approach, bone plate type, concurrent spay or neuter, anesthetic time, and surgical time on the likelihood of developing a surgical site infection and the likelihood of requiring an explantation separately (not controlling for signalment or other details). Though these 14 univariate analyses were used for hypothesis generation and screeining of variables for a multivariate model, if readers wish to assign significance based on the reported *p*-values, the Bonferroni-corrected *p*-value for significance with a type I error rate of 0.05 would be <0.004. Multivariate logistic regression was used to look at the combined effects of surgical time, concurrent spay or neuter, and surgical approach. The probabilities of requiring an explantation based on anesthetic and surgical time were plotted using the smoothed conditional density plot (“cdplot” function) of the data (Figure 1).

## 3. Results

### 3.1. Case data

A total of 116 greyhounds undergoing tarsal surgery were included in this study. The median age at the time of surgery was 37 months (range 10–30 months). Sixty-nine were male (52 intact, 17 castrated) and 47 were female (36 intact, 11 spayed). When evaluating the cause of tarsal injury, 105 (90.2%) were racing related injuries. The time from initial injury to surgery was available in 95 dogs, with a median of 3 days to surgery (range 0–1,461 days). Of 116 greyhounds undergoing tarsal surgery, 115 survived to discharge. One case was euthanized 4 days postoperatively due to incision dehiscence for which the owner declined therapy.

### 3.2. Tarsal injury description

Of the 116 greyhounds included in this study, 98 presented for a right tarsal injury (84.5%) and 18 presented with a left tarsal injury (15.5%). A total of 109 injuries were reported to be closed (94.0%) and 7 injuries were reported to be open (6.0%). Open fractures were Gustilo–Anderson grade I and involved the calcaneus. The most common tarsal injuries reported were CTB fractures (67/116; 57.8%), calcaneal fractures (66/116; 56.9%), and proximal intertarsal subluxation (40/116; 34.5%). Additional injuries diagnosed included fourth tarsal bone fractures (19/116; 16.4%), metatarsal bone five fractures (5/116; 4.3%), talar fractures (5/116; 4.3%), distal tibial articular fractures (2/116; 1.7%), and third tarsal bone fractures (1/116; 0.9%). Injury breakdown is further described in Table 2.

**Table 2:** Tarsal injuries diagnosed.

Injury	Number (%) of dogs
Central tarsal bone fracture	67 (57.8%)
Calcaneal fracture	66 (56.9%)
Talar fracture	5 (4.3%)
Third tarsal bone fracture	1 (0.9%)
Fourth tarsal bone fracture	19 (16.4%)
Fifth metatarsal fracture	5 (4.3%)
Joint subluxation/luxation	53 (45.7%)^a^
Distal tibial articular fracture	2 (1.7%)

a One of the fifty-three cases diagnosed with joint subluxation/luxation had multiple joints affected.

When evaluating injury combinations/configurations the most common injury presentation was CTB fracture with concurrent calcaneal fracture which was seen in 37/116 dogs (31.9%). Thirty-three dogs (33/116; 28.5%) were diagnosed with a single injury. Further evaluation of single injuries, two component injuries, three component injuries, and injuries involving greater than three components is provided in Table 3.

**Table 3:** Description of tarsal injury occurrences and combinations of injuries.

Injury	Number (%) of dogs
CTB fracture only	2 (1.7%)
Calcaneal fracture only	9 (7.8%)
T4 fracture only	1 (0.9%)
Joint subluxation/luxation only	21 (18.1%)
Injury with two components	
CTB and T4 fracture	7 (6.0%)
CTB and calcaneal fracture	37 (31.9%)
Calcaneus and talar fracture	1 (0.9%)
Calcaneus and T4 fracture	1 (0.9%)
CTB fracture and joint subluxation/luxation	7 (6.0%)
Calcaneal fracture with joint subluxation/luxation	7 (6.0%)
T3 fracture with joint subluxation/luxation	1 (0.9%)
T4 fracture and joint subluxation/luxation	2 (1.7%)
MT5 fracture and joint subluxation/luxation	1 (0.9%)
Distal tibial articular fracture and joint subluxation/luxation	2 (1.7%)
Injury with three components	
CTB, T4, and MT5 fracture	3 (2.6%)
CTB, calcaneus, and talar fracture	1 (0.9%)
CTB and T4 fracture with joint subluxation/luxation	1 (0.9%)
CTB and talar fracture with joint subluxation/luxation	2 (1.7%)
CTB and calcaneal fracture with joint subluxation/luxation	5 (4.3%)
Calcaneus and T4 fracture with joint subluxation/luxation	2 (1.7%)
Calcaneus and MT5 fracture and joint subluxation/luxation	1 (0.9%)
Injury with more than three components	
CTB, calcaneus, T4 and talar fracture	1 (0.9%)
CTB, Calcaneus, and T4 fracture with joint subluxation/luxation	1 (0.9%)

CTB, central tarsal bone; T3, third tarsal bone; T4, fourth tarsal bone; MT5, fifth metatarsal bone.

The 67 CTB fractures were further graded by the previously described classification system of Dee et al. (15) (Table 4). The most common CTB fracture grade recorded was a type IV CTB fracture (47/67; 70.2%). The remaining CTB fractures were classified as follows: type II in 3/67 cases (4.5%), type III in 2/67 cases (3.0%), type V in 12/67 cases (12/67; 17.9%), and plantar process fracture in 3/67 cases (4.5%).

**Table 4:** Central tarsal bone fracture classifications.

Central tarsal bone fracture grade	Number (%) of dogs with central tarsal bone fractures (*N* = 67)
I	0 (0%)
II	3 (4.5%)
III	2 (3.0%)
IV	47 (70.2%)
V	12 (17.9%)
Plantar process fracture	3 (4.5%)

The 66 calcaneal fractures were further described by their fracture configuration (Table 5). The most common configuration was a mid-body fracture with a fissure extending to the distal articular surface, seen in 36/66 cases (54.5%). The remaining calcaneal fractures were classified as follows: simple mid-body fracture in 3/66 cases (4.6%), comminuted fracture in 14/66 cases (21.2%), plantar tarsal ligament avulsion fracture in 10/66 cases (10/66; 15.2%), and base/articular injury in 3/66 cases (4.6%).

**Table 5:** Calcaneal fracture configuration.

Calcaneal fracture configuration	Number (%) of dogs with calcaneal fractures (*N* = 66)
Simple mid-body	3 (4.6%)
Comminuted	14 (21.2%)
Plantar tarsal ligament avulsion	10 (15.2%)
Mid-body with fissure extending to distal articular surface	36 (54.5%)
Base/articular injury	3 (4.6%)

Joint luxation/subluxation was diagnosed in 53 dogs (53/116; 45.7%). The level of joint luxation/subluxation can be seen in Table 6. The most commonly affected joint was the proximal intertarsal subluxation in 40/53 dogs (75.5%). The remaining joint instabilities were diagnosed as follows: tarsometatarsal subluxation in 6/53 cases (11.3%), talocrural luxation in 5/53 cases (9.4%), distal intertarsal subluxation in 1/53 cases (1.9%), and combined proximal intertarsal subluxation and talocalcaneal luxation in 1/53 cases (1.9%).

**Table 6:** Level of joint luxation/subluxation.

Joint level and severity	Number (%) of dogs with joint luxation/subluxation (*N* = 53)
Proximal intertarsal subluxation	40 (75.5%)
Distal intertarsal subluxation	1 (1.9%)
Proximal intertarsal subluxation and talocalcaneal luxation	1 (1.9%)
Tarsometatarsal subluxation	6 (11.3%)
Talocrural luxation	5 (9.4%)

### 3.3. Surgical procedure

Surgery was performed by a board-certified surgeon in 80 cases, a small animal surgery resident in 29 cases, and the surgeon was unspecified in 7 cases. A full list of all surgical procedures can be found in Table 7. The most commonly performed initial surgical intervention was a partial tarsal arthrodesis (50/116; 43.1%). Forty-four dogs underwent partial tarsal arthrodesis without additional procedures (44/116; 37.9%) and an additional 6/116 (5.2%) dogs underwent partial tarsal arthrodesis in combination with another surgical procedure. Joint level of arthrodesis was dependent on the injury configuration, but the joint most commonly undergoing arthrodesis was the calcaneoquartal joint accounting for 25/50 cases (50.0%). Additional joints undergoing arthrodesis included the proximal intertarsal joint (17/50; 34.0%) and tarsometatarsal joint (6/50; 12.0%). The joint level of arthrodesis was not recorded in 2 cases (Table 8). While under anesthesia for the initial surgical intervention of the tarsal injury, a total of 21/116 cases (18.1%) underwent concurrent elective sterilization. Following surgery, post-operative antibiotics were prescribed in 111 cases (111/116; 95.7%). The most commonly prescribed antibiotic was cephalexin (103 cases), followed by metronidazole (6 cases) and amoxicillin/clavulanic acid (5 cases). Ciprofloxacin, doxycycline, and enrofloxacin were used in one case each. Additionally, one patient received an unspecified oral antibiotic post-operatively.

**Table 7:** Initial surgical procedures performed.

Initial surgical procedure performed	Number (%) of dogs
Partial tarsal arthrodesis	44 (37.9%)
Partial tarsal arthrodesis with CTB screw fixation	4 (3.5%)
Partial tarsal arthrodesis with CTB screw fixation and lag screw fixation a calcaneal fracture	1 (0.9%)
Partial tarsal arthrodesis, fibular osteotomy with screw fixation, CTB screw fixation, and screw fixation for talar fracture	1 (0.9%)
Lateral bridging plate	14 (12.1%)
Lateral bridging plate with CTB screw fixation	25 (21.6%)
Lateral bridging plate with IM pin	1 (0.9%)
Lateral bridging plate with IM pin and CTB screw fixation	8 (6.9%)
Lateral bridging plate with calcaneal lag screw	2 (1.7%)
Lateral bridging plate with calcaneal lag screw, and CTB screw fixation	1 (0.9%)
CTB screw fixation	8 (6.9%)
Transarticular external skeletal fixator	1 (0.9%)
Calcaneal pin and tension band	1 (0.9%)
Talo-calcaneal screw	1 (0.9%)
Lateral malleolar pin and tension band	1 (0.9%)
Medial malleolar pin and tension band	1 (0.9%)
Lateral and medial pin and tension band with lag screw fixation of the caudal distal articular tibial fracture fragment	1 (0.9%)

**Table 8:** Level of joint arthrodesis.

Joint level	Number (%) of dogs (*N* = 50)
Calcaneoquartal joint	25 (50.0%)
Proximal intertarsal joint	17 (34.0%)
Tarsometatarsal joint	6 (12.0%)
Unknown	2 (4.0%)

### 3.4. Postoperative infection

The one case that was euthanized 4 days postoperatively was excluded from assessment of post operative infection. Culture and sensitivity were performed in 55/115 cases (47.8%). Culture was performed due to suspicion of infection based on physical examination, radiographic examination, or was performed at the time of explantation as a precautionary measure. Of those 55 cases, 21 cases had more than one culture performed over the course of treatment. Forty six of 55 (83.6%) cases had at least one positive culture, 7/55 (12.7%) had no growth on any of the cultures performed, and 2/55 (3.6%) had unknown results. Two cases were suspected to have active infection at the time of implant removal based on irregular periosteal new bone formation, soft tissue swelling, peri-implant lucency, and response to empirical antibiotic therapy, but no culture and sensitivity was performed. Based on positive culture, the overall SSI rate was 40.0% (46/115). Of these cases, 6 presented with an open fracture and were therefore not considered a clean procedure. Excluding these 6 cases, the overall SSI rate for clean procedures in this study was 36.7% (40/109).

When evaluating all cases that had positive culture results reported in the medical record (including cases with multiple cultures over time), the most commonly isolated organism was *Staphylococcus pseudintermedius*, which was identified in 35 cases. The remaining isolates are available for review in Table 9. For cases with available sensitivity results 34.3% (12/35) of *S. pseudintermedius* cases showed evidence of methicillin resistance (MRSP).

**Table 9:** Bacterial culture isolates.

Isolate	Number of times isolated
*Staphylococcus pseudointermedius*	35
*Pseudomonas aeruginosa*	6
*Actinomyces* sp.	4
*Enterobacter cloacae*	4
*Enterococcus faecium*	3
*Escherichia coli*	4
*Pasteurella multocida*	3
*Streptococcus* sp.	3
*Bacillus* sp.	2
*Enterococcus hirae*	2
*Peptpstreptococcus* sp.	2
*Staphylococcus aureus*	2
*Staphylococcus epidermis*	2
*Staphylococcus* sp. (unidentified)	2
*Acinebacter* sp.	1
*Arcanobacterium* spp.	1
*Bacteriodies* sp.	1
*Enterococcus faecalis*	1
*Enterococcus* sp.	1
*Porphyromonas* sp.	1
*Proteus mirabilis*	1

Factors associated independently with an increased likelihood of surgical site infection included performing both a medial and lateral approach compared to a single approach {*p* = 0.007; odds ratio = 2.95, 95% confidence interval [CI] (1.35, 6.59)}, concurrent spay or neuter [*p* = 0.030; odds ratio = 2.95, 95% CI (1.13, 8.16)], the use of a locking plate [*p* = 0.013; odds ratio = 3.45, 95% CI (1.35, 9.72)], and increased anesthetic/surgical time [*p* = 0.023; for each additional 20 min odds ratio = 1.17, 95% CI (1.03, 1.36)]. None of these were significant at the Bonferroni-corrected *p*-value cutoff of 0.004. All other variables were not associated with increased odds of SSI. In dogs that received a bone plate as part of surgical repair, nonlocking plates had lower odds of developing a surgical site infection (reported above) compared to locking plates. Compared to the entire cohort (patients receiving any type of fixation), patients receiving a locking plate were at a significantly higher risk of developing a surgical site infection [*p* = 0.0002; odds ratio = 5.76, 95% CI (2.37, 15.71)]. When controlling in a multivariate model for dogs that had both medial and lateral approaches and a concurrent spay/neuter together and accounting for surgical time, the only significant effect on developing a surgical site infection was having a medial and lateral approach [*p* = 0. 038; odds ratio = 2.43, 95% CI (1.05, 5.70)]. Seven of 116 (6.0%) injuries were classified as open at the time of presentation. Of these 7 cases, one was the aforementioned case that was euthanized 4 days postoperatively. The remaining 6 were all diagnosed with an SSI via positive culture postoperatively. All these cases presented with a calcaneal fracture as a component of their tarsal injury.

### 3.5. Explantation

Of the cases that survived to discharge, explantation was performed in 59/115 (51.3%) dogs. The most common recorded cause for explanation was concern for infection (44/59; 74.6%). The odds of explantation for animals with an SSI was 49.69 times the odds of explantation for those without a surgical site infection (*p* < 0.000). A total of 10/59 cases (16.9%) were explanted for a reason other than infection, including implant migration, implant exposure, suspected irritation of soft tissue by implants, planned external fixator removal, and implant failure. For the remaining 6/59 cases (10.1%), the underlying reason for explantation could not be discerned from the available medical record. Median time to explantation was 159 days following surgery (range 40–1,728 days).

Patients receiving an arthrodesis of the proximal intertarsal joint were less likely to have an explantation compared to patients that did not undergo arthrodesis [*p*-value = 0.011; odds ratio = 0.21, 95% CI (0.05, 0.65)] on univariant analysis.

Patients with calcaneal fractures (all types) had higher odds of requiring an explantation compared to patients with no calcaneal fracture [*p* = 0.023; odds ratio = 2.40, 95% CI (1.14, 5.17)]. Specifically, patients diagnosed with mid-body calcaneal fractures with fissures extending to the distal articular surface had a higher risk of requiring an explantation compared to those cases without a calcaneal fracture [*p*-value = 0.008; odds ratio = 3.41, 95% CI (1.40, 8.70)]. This would not be considered significant when using the conservative Bonferroni-corrected *p*-value cutoff of <0.004.

Surgical procedure factors independently associated with an increased risk of requiring explantation in univariate analysis included concurrent medial and lateral surgical approaches, concurrent spay or neuter, and the use of locking plates. The odds of explantation for dogs receiving both a medial and lateral surgical approach to the tarsus were 3.24 times the odds as those receiving only a medial or lateral approach [*p* = 0.004, 95% CI (1.49, 7.33)]. Concurrent spay or neuter was associated with an increased odds of requiring an explantation [*p* = 0.006; odds ratio = 5.04, 95% CI (1.71, 18.54)]. In patients having received a bone plate as part of surgical repair, nonlocking plates had lower odds of requiring an explantation [*p* = 0.0030, 95% CI (0.12, 0.72)] compared to locking plates. Compared to the entire cohort, patients receiving a locking plate were at a higher risk of requiring an explantation [*p* = 0.009; odds ratio = 3.38, 95% CI (1.38, 8.69)]. In addition, there was a greater odds of explantation associated with an increasing number of locking screws both proximally [*p* = 0.064; odds ratio = 1.38, 95% CI (0.99, 1.96)] and distally [*p* = 0.024; odds ratio = 1.53, 95% CI (1.08, 2.27)]. As with SSIs, none of these factors examined in univariate statistical screening would be considered significantly associated with the risk of explantation using the conservative Bonferroni-corrected *p*-value cutoff of <0.004.

There was a relatively regular progression of increasing risk of explantation with increasing surgical and anesthetic time (Figure 1). For each additional 20 min of surgery time, the odds ratio of explantation was 1.17 [*p* = 0.023, 95% CI (1.03, 1.36)]. There was a spike in probability of explantation with anesthetic times less than 180 min and a dip in probability of explantation with surgical times greater than 240 min. When controlling for dogs that had both medial and lateral approaches and a concurrent spay/neuter together, the only significant effect on explanation was having both a medial and lateral approach [*p* = 0.049; odds ratio = 2.48, 95% CI (1.01, 6.25)].

### 3.6. Additional complications

A total of 38 additional complications were noted in 32/116 (27.6%) of cases. These complications ranged in severity with varying interventions required. A full list of additional complications and the number of patients with these complications is reported in Table 10.

**Table 10:** Additional complications.

Additional complication	Number of dogs
Implant exposure	15
Re-luxation of joint or residual instability requiring revision surgery	5
Implant failure	4
Suboptimal fracture reduction requiring surgical revision	3
Suboptimal fracture or joint reduction with minimal clinical impact and no surgical intervention required	2
Refracture with amputation elected	2
Stripping of the screw during CTB repair	1
Inappropriate directing of the screw used for CTB fracture reduction	1
Collapse of the fourth tarsal bone during healing leading to tarsal valgus	1
Collateral instability due to implant migration	1
Refracture requiring revision surgery	1
Progressive osteomyelitis with amputation elected	1
Euthanasia 4 days postoperatively due to incisional dehiscence and owner reluctance to move forward with additional therapy	1

## 4. Discussion

Tarsal injuries in greyhounds present significant management challenges due to their thin skin (19) and the minimal soft tissue coverage associated with the distal limb (20). Previous reports have examined the frequency of tarsal injuries in the racing greyhound with the majority involving fractures of the CTB (7). Of the 116 greyhounds evaluated in this study, CTB fractures were the most frequent injury component with calcaneal fractures and proximal intertarsal subluxation also reported with high frequency (57.8%, 56.9%, and 34.5% respectively). The most common surgical repair in this group of patients was a partial tarsal arthrodesis, most frequently performed at the level of the calcaneoquartal joint. A variety of postoperative complications were recorded in this cohort of patients. Of significant clinical importance, the overall SSI rate was noted to be higher than previously reported for orthopedic procedures allowing us to accept our hypothesis.

Of the 115 greyhounds that survived to discharge, 40.0% were diagnosed with an SSI via positive bacterial culture. Of these, 6 cases presented with an open fracture and were therefore not considered a clean procedure. Excluding these 6 cases, the overall SSI rate for clean procedures in this study was 36.7% (40/109). The SSI rate for clean orthopedic surgeries has been previously reported to range from 3.3% to 7.1% (21–25). Greyhounds in this study undergoing a clean orthopedic procedure were almost 5 times more likely to develop a surgical site infection compared to previously reported literature. Even when compared to the tibial plateau leveling osteotomy, an orthopedic procedure that has a surgical site infection rate as high as 15.8% (14), the reported infection rate in this study is more than double what has been historically reported. Armstrong et al. (26) reported a postoperative SSI rate of 10.7% in nonracing dogs with CTB injuries. We postulate that the reason for the higher SSI rate in our study is primarily secondary to patient population. Greyhounds have thinner skin which is more prone to damage compared to other breeds (19) increasing the risk of incision dehiscence, bandage associated morbidity, implant exposure, and SSI. Additionally, greyhounds have a paucity of subcutaneous tissue compared to other dog breeds. Previous literature has shown that subcutaneous tissue plays an important role in cutaneous wound healing (27). In a study by Bohling et al. (27) the removal of the subcutaneous tissue caused a significant reduction in perfusion of open wounds. This demonstrated the importance of the subcutis in 2nd intention healing which is elected on occasion in greyhounds when excessive tension would be noted with incisional closure (27). Interestingly, this is in direct opposition to several studies in human literature that have found that increased soft tissue thickness/coverage (>5 cm) in patients undergoing total hip arthroplasty (28), or repair of patellar fractures (29) is associated with an increase in infection rate.

In this study, we report the SSI rate for surgical repair of tarsal injuries in greyhounds. In people, the reported SSI rate for clean orthopedic procedures is 0.5% to 6.5% (30). While the specific SSI rate reported for foot and ankle surgery is similar at 1.0% to 5.3% (31–36), it is suspected that it might actually be higher due to reduced ability to mitigate bacterial load with surgical preparation compared to more superior (proximal) surgical sites (36, 37). This challenge in surgical preparation holds true for dogs, and may help explain why our SSI rate was higher than previously reported rates in clean veterinary orthopedic procedures. Additionally, higher bacterial loads on dogs’ skin (compared to humans) has been demonstrated in studies evaluating skin asepsis protocols (35). This is believed to be at least in part due to the presence of fur, low hygiene (bathing) frequency, and contact with a more contaminated environment (35).

Dogs undergoing both a medial and lateral surgical approach were significantly more likely to develop an SSI and require an explantation. In this study, 38.8% of dogs (45/116) underwent bilateral approaches for surgical repair of their orthopedic disease. Of these 45 dogs, 53.3% (24/45) were diagnosed with an SSI and 68.9% (31/45) required explantation. Cases receiving bilateral approaches were reported to have longer anesthesia and surgery times on average compared to dogs undergoing a single surgical approach. Despite the longer anesthesia and surgical times, multivariate statistical analysis revealed that undergoing both a medial and lateral surgical approach retained significant impact on SSI rate and need for explantation when controlling for the extended anesthesia/surgery time. We hypothesize that this increased likelihood of developing an SSI and need for explantation is likely secondary to additional tissue trauma and increased wound tension. When both medial and lateral approaches are performed, the thin skin and sparsity of subcutaneous tissue of greyhounds often poses a challenge with performing incisional closure over implants with some patients having incisions that are left partially open to avoid a tourniquet effect from excessive tension. We suspect that these challenges could contribute to an increase in wound dehiscence and implant exposure. In fact, of the 15 dogs that were reported to have implant exposure, 66.7% (10/15) had both a medial and lateral surgical approach performed. While this finding is of note, the need for both medial and lateral approaches is often unavoidable with specific injury conformations (CTB fractures with concurrent calcaneal fractures, CTB fractures with proximal intertarsal instability, etc.). When bilateral approaches are required, the surgeon should pay utmost attention to minimizing soft tissue trauma and diligent monitoring for SSIs postoperatively.

Dogs that received an elective spay or neuter at the time of surgery were more likely to develop an SSI and require an explantation using univariate analysis. Of the 21 dogs receiving an elective castration at the time of their orthopedic surgery, 61.9% (13/21) were diagnosed with an SSI and 81.0% (17/21) required an explantation. Dogs receiving elective castration were reported to have longer anesthesia and surgery times than those not receiving castration. A previous study in human literature found that undergoing multiple surgical procedures was associated with a significant increase in risk of developing a surgical site infection although this was mainly caused by prolonged duration of surgery (38, 39). However, in our univariate analysis, the effect of dogs undergoing elective castration had a higher impact on developing an SSI and need for explantation than anesthetic or surgery time. We hypothesize that the additional surgical procedure increased the surgical field and may have increased the risk of breaches in sterility. Additional studies are needed to elucidate these findings. Regardless, the potential for increased risk of surgical site infection and explantation should be considered when performing additional surgical procedures concurrently with orthopedic surgical intervention in greyhounds with tarsal injuries.

Longer anesthetic and surgical times were also found to have an increased risk of explantation in our univariate analysis. For each additional 20 min of surgery time, the odds ratio of explantation was 1.17. There was a spike in probability of explantation with anesthetic times less than 180 min and a dip in probability of explantation with surgical times greater than 240 min. This may be explained by the low number of cases with an anesthetic time less than 180 min (16/116) or a surgery time greater than 240 min (2/116). With the exception of these two deviations, there was a relatively regular progression of increasing risk of explantation with increasing anesthetic and surgical time. Increased duration of surgery has been previously reported as a risk factor for the development of SSI (which was associated with explantation in our study using univariate analysis) (40–44). It has been proposed that increased surgical time increases the amount of environmental contamination in surgical wounds (45). In addition to increased exposure to environmental contamination, increased surgical time may be associated with more complicated procedures and progressive wound desiccation allowing for increased ability of bacteria to enter the surgical field (46). In agreement with previous studies, our findings support that time under anesthesia and duration of surgery should be limited to minimize the risk of SSI and subsequent explantation.

Patients diagnosed with calcaneal fractures had 2.4 times the odds of requiring an explantation compared to patients without calcaneal fractures using univariate analysis. Additionally, of the 66 calcaneal fractures reported, 57 sustained additional tarsal injury, therefore increasing dissection and surgical time if the other injuries required specific surgical treatment. This may have increased the risk for wound dehiscence or SSI. Patients with calcaneal fractures commonly receive a bone plate as a component of the surgical intervention. The use of a locking plate was found to be associated with an increased risk of developing a surgical site infection and requiring explantation using univariate analysis. When directly comparing the use of locking and nonlocking plates, nonlocking plates show a decreased odds of developing an SSI. This relationship proved similar when evaluating the risk of requiring an explantation. The increased risk of developing an SSI when a locking plate was used (compared to a non-locking plate) is surprising. Locking bone plates and screws have been shown to reduce infection rates compared to conventional dynamic compression plates (DCPs) in previous literature (47–49). This protective effect was suggested to be secondary to a lack of perfusion disruption to the cortical bone underlying the plate (compared to DCPs), decreased incidence of inflammation from loosening hardware, and faster bone healing due to a more stable fixation (47). One explanation for our findings may be the lack of requirement for bone contact throughout the length of a locking plate, resulting in a prouder implant that may wear through the thin tissues of greyhounds, resulting in reduced soft tissue coverage and in some cases, implant exposure. An alternative explanation is that in our study, 72.3% of dogs undergoing plating received a locking plate (73/101). This resulted in unequal representation in the data between locking and nonlocking plates.

Patients undergoing arthrodesis of the proximal intertarsal joint had a decreased odds of requiring an explantation on univariate analysis. This finding is interesting as other publications have found explantation rates of up to 21% following partial tarsal arthrodesis (50) and 12.5% following tarsal arthrodesis (51). In this patient population, factors that increased risk of explantation were all surgical interventions requiring significant soft tissue disruption and manipulation. Partial tarsal arthrodesis requires less soft tissue manipulation compared to repair of complex calcaneal fractures. Additionally, proximal tarsal arthrodesis is performed through a single surgical approach compared to a bilateral approach, which is used to address CTB fractures with concurrent calcaneal fractures, the most common injury presentation in our study. Given that this association was found with the univariate analysis, it may be confounded by the effect of surgical approach, so further exploration is warranted.

Limitations to this study are primarily related to the retrospective nature which required a reliance on medical records. This design leads to the presence of confounding factors such as incomplete information regarding clinical signs and complications. Multiple doctors were scrubbed into surgery, and it was not always clear which doctor (and which experience level of doctor, i.e., resident vs. boarded surgeon) performed the procedure or if primary responsibility shifted throughout. Additionally, at the time of last follow up, several patients had a lack of complete healing on radiographs. This limitation in follow-up could have led to complications being missed in some patients. In some cases, these animals may have presented to other veterinarians for care related to the operated limb in the years following which may have led us to underestimate the infection rate and subsequent explant rate for this population of dogs. Lastly, we did not incorporate a control group into this study so we cannot report whether other dog breeds with tarsal injuries would have same complications and complication rates as greyhounds in our care.

It should be noted that our patient population is biased towards more severe, career ending racing injuries. Our institution receives referral for severe racing injuries from a regional racetrack such as comminuted fractures, long-bone fractures, severe intertarsal joint luxation, and calcaneal fractures, whereas greyhounds with CTB fractures that do not include subluxation typically undergo cast immobilization under local veterinary clinic care. This decision is made at the discretion of the overseeing Racing Commission State Veterinarian (52). In 2021 (the most recent report available at the time of this publication), a total of 220 track injuries (not limited to orthopedic injuries) were reported at a single race track. Of these 220 cases, only 21 were referred to our clinic (9.6%). Therefore, our findings cannot and should not be expected to apply to all greyhound tarsal injuries.

The greyhound racing industry has significantly evolved over the course of the last decade. At the time of this publication, only two greyhound racing tracks remain active in the United States and only 7 countries have active greyhound racing programs. As racing greyhounds become less prevalent in the veterinary field, it would be interesting to document the occurrence of tarsal injuries in non-racing or retired greyhounds. This point of investigation could be considered for future research.

We concluded that the SSI rate in this population of greyhounds undergoing surgical repair of closed tarsal injuries is higher than previously reported for clean orthopedic procedures. All greyhounds that presented with open fractures went on to develop a SSI. Additionally, over half of greyhounds undergoing ORIF of tarsal injuries will require explantation of the surgical implant. Although they can do well clinically following explantation, this increases the cost to the owner and the recovery period for the dog. Factors needing further exploration for their association with an increased rate of SSI and explantation included concurrent elective sterilization, calcaneal fractures, use of locking plates, and increased duration of anesthesia and surgery. The information in this study can help guide practitioners’ clinical expectations and client communications when undertaking these challenging cases. Practitioners performing surgical intervention for tarsal injuries in greyhounds should be prepared for the high likelihood that explantation will be required following healing, particularly those requiring both a medial and lateral surgical approach. Additionally, anesthesia and surgery time should be minimized in these cases due to the possible correlation with increased risk of SSI and explantation. Future consideration should be given to explore alternative surgical techniques for managing these cases such as minimally invasive approaches or the use of external skeletal fixation with the goal of lowering the SSI and explantation rate in this patient population.

## Data availability statement

The raw data supporting the conclusions of this article will be made available by the authors, without undue reservation.

## Ethics statement

Ethical review and approval was not required for the animal study because this was a retrospective study.

## Author contributions

NK and JD contributed to the conception and design of the study. NK, JD, AW, SJ, ST, MB, BC, KS, and CF contributed to the data collection and organization for the study. MB wrote the first draft of the manuscript. All authors contributed to the article and approved the submitted version.

## Conflict of interest

The authors declare that the research was conducted in the absence of any commercial or financial relationships that could construed as a potential conflict of interest.

## Publisher’s note

All claims expressed in this article are solely those of the authors and do not necessarily represent those of their affiliated organizations, or those of the publisher, the editors and the reviewers. Any product that may be evaluated in this article, or claim that may be made by its manufacturer, is not guaranteed or endorsed by the publisher.
